# New Models of Emergency Prehospital Care That Avoid Unnecessary Conveyance to Emergency Department: Translation of Research Evidence into Practice?

**DOI:** 10.1155/2013/182102

**Published:** 2013-06-02

**Authors:** Helen Anne Snooks, Mark Rhys Kingston, Rebecca Elizabeth Anthony, Ian Trevor Russell

**Affiliations:** Centre for Health Information Research and Evaluation (CHIRAL), Institute of Life Science, College of Medicine, Swansea University, Singleton Park, Swansea SA2 8PP, UK

## Abstract

*Background*. Achieving knowledge translation in healthcare is growing in importance but methods to capture impact of research are not well developed. We present an attempt to capture impact of a programme of research in prehospital emergency care, aiming to inform the development of EMS models of care that avoid, when appropriate, conveyance of patients to hospital for immediate care. *Methods*. We describe the programme and its dissemination, present examples of its influence on policy and practice, internationally, and analyse routine UK statistics to determine whether conveyance practice has changed. *Results*. The programme comprises eight research studies, to a value of >**£**4 m. Findings have been disseminated through 18 published papers, cited 274 times in academic journals. We describe examples of how evidence has been put into practice, including new models of care in Canada and Australia. Routine statistics in England show that, alongside rising demand, conveyance rates have fallen from 90% to 58% over a 12-year period, 2,721 million fewer journeys, with publication of key studies 2003–2008. *Comment*. We have set out the rationale, key features, and impact on practice of a programme of publicly funded research. We describe evidence of knowledge translation, whilst recognising limitations in methods for capturing impact.

## 1. Background

The gap between the production of research evidence and implementation into routine clinical practice has been well acknowledged and has been referred to as the second translational gap; the first gap is that between laboratory science and clinical research [1]. With increasing recognition of the importance of not only carrying out research but also of ensuring that research findings are taken up and used by those making health care policy and providing health care, researchers and research funders are now paying more attention to dissemination, particularly active forms which have the ability to influence care delivery, and also to capturing the impact of research [2–4].

In the field of emergency care, research evidence to underpin care has been criticised both for its scarcity and quality [5]. In the prehospital setting these concerns are even more acute [6–8]. Emergency prehospital care is a field without a strong academic tradition, but patient volume is high and outcomes are linked to responses provided by emergency medical systems (EMS). In this growing field, demonstrating impact in practice is fundamental to the continued attraction of research funding, building of research skills and culture, and thus a high-quality evidence based to inform future policy and practice.

With sustained increases in demand for emergency prehospital care across the developed world, current systems are unable to maintain services that traditionally respond to all emergency calls to the ambulance service with a paramedic staffed patient carrying vehicle travelling on lights and sirens, and with a default of conveyance to an emergency department (ED) for medical care unless the patient refused to travel. Researches focussing on the needs and outcomes of patients for whom emergency (999) calls to the ambulance service are made have shown that a substantial proportion of these patients (up to 52%) [11, 12, 15, 28] do not need immediate medical care, but that triage systems at the despatch centre and on scene that identify patients for self- or community-based care carry significant safety risks [15–17, 22]. Unnecessary transportation can also be an issue for patients who have little or no chance of survival [29].

In this paper we describe a programme of research related to the development and implementation of new models of care that allow ambulance services to offer alternatives to the traditional response and to safely reduce conveyance of patients to ED and present data that demonstrate the impact of this research.

## 2. Summary of Research Programme

Supported by over *£*4 m in research grants, the programme of work includes studies that have followed the UK's Medical Research Council's Framework for the Development and Evaluation of Complex Interventions [30, 31] and comprises reviews of existing research and practice and experimental research, including randomised controlled trials (Table 1). Research findings indicate and describe the problem [9–13] and then the costs and effectiveness of alternatives to current practice [14–19, 22]. Study findings have been widely disseminated to generic and specialist audiences through publication in peer reviewed and practitioner journals, as well as at conferences at local, national, and international levels. The research team works closely with prehospital care providers and policy makers in the UK but does not follow a formal knowledge transfer strategic approach.

## 3. Impact on Policy and Practice: Knowledge Transfer

### 3.1. Methods

We tracked citations using Google Scholar, undertook extensive electronic searching of policy documents, and gathered ad hoc information related to service developments in which studies from this programme of work were cited. We analysed routine national data provided by all individual services as part of their required performance statistics for the period before and since publication of findings from studies within the programme.

### 3.2. Results

Papers reported in Table 1 have been cited in academic journals 274 times to date. An influential systematic review of 999 alternatives for the UK Department of Health (2005) draws heavily upon the work of the research team and has gone on to influence guidance emanating from statutory UK bodies [32–34]. Nontransport (to ED) guidelines from the Ambulance Service Association and Department of Health, which cite elements of this work, have been widely adopted, as have “Treat and Refer” protocols.

Enhanced telephone triage has been adopted across the UK ambulance service providers, in line with the recommendations of the Department of Health and the Ambulance Service Association—both of which respond to work published within this programme. Through correspondence and desktop reviews we are aware of similar service models having been adopted internationally, in Canada, for example, and in South Australia, where the Ambulance Service was able to report financial savings following implementation across the state of Victoria, having cited findings from the Telephone Advice Study [13–15], in their business case.

*“Prior to 2003 we sent an ambulance to all calls received via the “000” ambulance emergency call centre. Professor Snooks work, including the evidence of very high caller satisfaction and very few adverse events from referrals instead of conveyance, was used to show the need for a referral service at point of call. In the year following implementation we were able to show cost savings and have now fully implemented the service, and the model is being rolled out across Australia.”*Senior Research Fellow, Ambulance Victoria, Australia.**

*“We have used Professor Snooks published work [on pre hospital clinical decision making tools] to inform policies in Nova Scotia and Alberta. There are similar challenges being faced in the UK and Canada.”*Senior Performance Strategist, Alberta Health Services Emergency Medical Services. **

**Figure 1:**
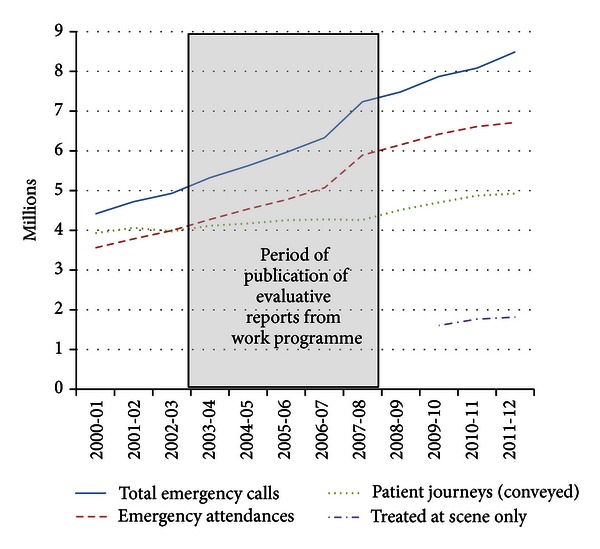
Ambulance Service Summary Statistics (a), England, 2000–2012.

**Figure 2:**
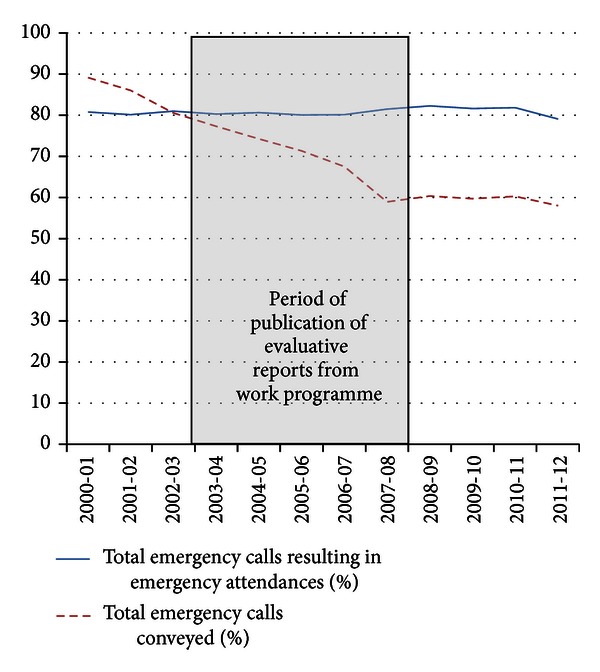
Ambulance Service Summary Statistics (b), England, 2000–2012.

Evidence of the impact of the work in prehospital care can be seen in conveyance rates—90% of emergency calls in England resulted in hospital conveyance in 2000 compared to 58% in 2012 (see Figures 1 and 2)—equivalent to 2,721 million fewer journeys.

## 4. Discussion

### 4.1. Key Points

Findings from this programme of work have consistently highlighted the need for alternatives to routine conveyance of 999 patients to ED and the team have developed, implemented, and tested a range of approaches to improving and providing evidence about the quality, safety, and cost effectiveness of care.

Working collaboratively with the NHS and policy makers, lessons from the programme of work have been disseminated widely in peer-reviewed articles, policy literature, international conferences, and through personal invitations to visit service providers.

Nationally and internationally, evidence from this programme of work has been cited in policy documents and in service developments, including the provision of telephone advice and Treat and Refer protocols.

In the face of consistent increases in demand for the 999 emergency ambulance service in the UK and internationally, we have demonstrated evidence of falling conveyance rates and an increasing proportion of patients treated at scene in England since the publication of our findings.

### 4.2. Strengths and Limitations

Methods for capturing impact of research are not well developed and include a variety of approaches [35, 36]. In this under researched area, policy documents are often consensual rather than based on evidence and citation of underpinning research is rare.

In this paper we have described the scope, characteristics, and impact of a research programme in emergency prehospital care. For inferences about impact on practice we have had to rely on citations and ad hoc reports of service innovation alongside routine statistics related to emergency demand and treatment. Citations are recognised as a weak indicator of real impact [36]. Routine data are reliable but observational.

We are conscious that there are other potential causes for these changes. In an ideal or planned world with multiple indicators and well-defined interventions, the statistical technique of interrupted time series can draw stronger conclusions about cause and impact, as in the Respect trial [37, 38]. In the real world inferences about causation are more difficult.

### 4.3. Implications

Demonstration of impact of research is increasingly important in times when resources are scarce and competition is heavy. Research funders and researchers are under pressure to report impact but methods are underdeveloped. Policy and treatment guidelines often lack transparent underpinning research evidence. Measuring impact is our only way of capturing knowledge transfer from research evidence to patient care.

Against this setting we have attempted to set out the rationale, key features, and resulting impact on practice of a programme of research funded through the public purse in the UK. We argue that findings have been influential at national and international levels although we recognise that the rigour of methods for identifying and attributing impact is not as high as in the traditional “gold standard” RCT.

## Figures and Tables

**Table 1:** Summary of studies and outputs included in research programme.

Study and funding	Research focus	Research sites, patient population	Research design	Key messages and references
Epidemiology of emergency ambulance calls South West Regional Health Authority R&D 1996–1999	To increase the knowledge base regarding demand for emergency medical services and the factors influencing demand	Wiltshire All 999 callers	Observational study and review of evidence	(i) Demand for emergency ambulances rose by 72% in Wiltshire between 1988 and 1996 with no evidence of an increase in GP placed calls [9] (ii) Calls to emergency ambulance service rising (1993–5 England 8% increase, varying from <1% in Durham to 20% in West Country) [10] (iii) Determinants of ambulance usage little researched [10] (iv) Although rates vary (from 19–52%), inappropriate use of emergency ambulances found consistently [11, 12] (v) Research to developand evaluate alternatives to current 999 system urgently needed

Telephone Advice Study NHS Primary and Secondary Care Interface R&D programme 1996–1999	To assess the safety of nurses and paramedics offering telephone assessment, triage, and advice as an alternative to immediate ambulance despatch for emergency ambulance service callers classified by non-clinical call takers as presenting with “non-serious” problems	London, West Midlands 999 callers triaged in ambulance call centre as low priority	Quasi experimental, shadow trial	(i) 999 alternatives that leave patients at home instead of taking them to hospital carry safety risks [13] (ii) Telephone assessment in the ambulance call centre can identify patients who may not need to be attended by emergency ambulance [14] (iii) No serious safety issues were found in this shadow trial [15] (iv) Full randomised trial of clinical and cost effectiveness of telephone advice for non-urgent 999 callers warranted [13–15]

Minor Injuries Units study NHS North Thames Regional Health Authority R&D programme 1999–2002	To evaluate triage and transportation to a minor injury unit (MIU) by paramedics	London, Surrey Patients with minor injuries attended by 999 ambulance	Randomised controlled trial	(i) Trial results reported by treatment received due to low study compliance [16] (ii) MIU usage was low (10% of eligible patients) [16] (ii) Patients taken to MIU were more likely to rate their care as excellent, with emergency ambulance episode and total emergency episodes considerably shorter for these patients [16]

Treat and Refer study NHS North Thames Regional Health Authority R&D programme 1999–2002	To develop and evaluate “Treat and Refer” protocols for paramedics, allowing them to leave patients at the scene with onward referral or self-care advice as appropriate	London Patients who may not need to be taken to Emergency Department (ED) for immediate care	Quasi experimental	(i) With interest in developing alternatives high, few services (2/42) had formally audited non-conveyance or (9/42) put new models of care into practice. Only 3 services had carried out any evaluation of these initiatives [17] (ii) 23 protocols were developed for the face to face assessment and care of 999 patients who may not need to be taken to ED for treatment [18] (iii) 17 were used, for 40% of intervention group patients (*n* = 101/251), with falls protocol used much more frequently than any other (*n* = 57) [18] (iv) Conveyance rates were unaffected, but protocols were found to be safe and patient satisfaction was high [18] (v) There was consensus at the end of the project that Treat and Refer protocols should be introduced across the service, but crews reported feeling unsupported to change practice [19] (vi) Further research needed at multiple sites barriers to implementation need to be addressed [17–19]

Fit to be Left NSF for Older people R&D programme 2003–2006	To design, develop, implement and evaluate a tool designed to support ambulance staff to make consistent and formalised decisions concerning the conveyance of older people who have fallen	London Patients aged 65+ attended by a 999 ambulance following a fall	Observational, quasi experimental	(i) Patients left at home by their attending crew following a fall were at high risk of a further fall, 999 attendance, ED contact and death within 2 weeks [20] (ii) Decisions to leave older people at home following a fall were complex and multi factorial [21]

Non serious 999 calls managed by nurse advisers by telephone NHS Service Delivery and Organisation R&D programme, 2002–2005	An evaluation of the costs and benefits of transferring some low priority 999 calls to NHS Direct nurse advisers for further assessment and advice	South Wales, Thames Valley Greater Manchester 999 callers with problems assessed in ambulance call centre as non-urgent	Randomised controlled trial	(i) Transferring non-urgent 999 calls for further advice and assessment provides a safe and cost-effective service for some calls [22] (ii) Almost half of calls transferred were returned to the ambulance service for an ambulance response indicating that, although non-urgent, many of these calls are for patients who need transport or some form of face to face assessment [22] (iii) Further research required todevelop and evaluate models of care that suit the range of 999 callers [22]

Non conveyance Wales Office of R&D, 2004–2006	Exploration of ambulance crew members' attitudes towards clinical documentation and non-conveyed patients	South Wales Patients left at home following an attendance by emergency ambulance	Qualitative study, focus groups	(i) Decision making complex for two reasons: capacity of patients to make decisions and input of patients, friends, family and ambulance crew [23] (ii) Mismatch between policy and practice needs to be addressed through research [23] (iii) Low rates of clinical documentation for 999 patients not taken to ED pose a litigation risk but the process is not valued by clinical staff or adequately audited by managers [24]

Paramedic Practitioner Older People Study The Health Foundation 2003–2006	To evaluate the safety, effectiveness and cost effectiveness of clinical decisions made by Paramedic Practitioners operating within the new service compared with standard practice of EMS transfer and ED treatment	Sheffield 999 patients aged 65+	Randomised controlled trial	(i) Patients in the intervention group were less likely to attend ED, require hospital admission within 28 days, experienced a shorter episode of care time, and were more likely to report being highly satisfied with their care, with no difference in 28 day mortality [25] (ii) 219/2025 patients attended ED within 7 days of their index call, in which 16 (0.8%) were judged to have received suboptimal care. No difference was found in rate between intervention and control arms [26] Paramedics with extended skills can provide a safe, clinically and cost effective alternative to standard ambulance transfer and treatment in an ED for elderly patients with acute minor conditions [25–27]
